# Hedgehog and PI3K/Akt/mTOR Signaling Pathways Involvement in Leukemic Malignancies: Crosstalk and Role in Cell Death

**DOI:** 10.3390/cells14040269

**Published:** 2025-02-13

**Authors:** Mariaconcetta Sicurella, Marica De Chiara, Luca Maria Neri

**Affiliations:** 1 Department of Environmental Sciences and Prevention, University of Ferrara, 44121 Ferrara, Italy; scrmcn@unife.it; 2 Department of Translational Medicine, University of Ferrara, 44121 Ferrara, Italy; dchmrc@unife.it; 3 LTTA-Electron Microscopy Center, University of Ferrara, 44121 Ferrara, Italy

**Keywords:** Hedgehog (Hh) signaling, PI3K/Akt/mTOR pathway, leukemia, autophagy, apoptosis

## Abstract

The Hedgehog (Hh) and PI3K/Akt/mTOR signaling pathways play a pivotal role in driving the initiation and progression of various cancers, including hematologic malignancies such as acute lymphoblastic leukemia (ALL), acute myeloid leukemia (AML), chronic myeloid leukemia (CML), and chronic lymphocytic leukemia (CLL). These pathways are often dysregulated in leukemia cells, leading to increased cell growth, survival, and drug resistance while also impairing mechanisms of cell death. In leukemia, the Hh pathway can be abnormally activated by genetic mutations. Additionally, the PI3K/Akt/mTOR pathway is frequently overactive due to genetic changes. A key aspect of these pathways is their interaction: activation of the PI3K/Akt pathway can trigger a non-canonical activation of the Hh pathway, which further promotes leukemia cell growth and survival. Targeted inhibitors of these pathways, such as Gli inhibitors and PI3K/mTOR inhibitors, have shown promise in preclinical and clinical studies.

## 1. Introduction

The Hedgehog pathway, initially recognized for its role in embryonic development, has gained significant attention in recent years for its involvement in tumorigenesis and its contribution to drug resistance in various cancers, including leukemia. Emerging evidence suggests that this pathway may interact with the PI3K/Akt/mTOR pathway, which is known as a modulator of cell growth, metabolism, and survival, and its dysregulation is often associated with leukemia, where it drives clonal expansion of malignant cells.

This interaction plays a key role in promoting resistance to cell death, and it is essential to the creation of targeted therapies. This review aims to explore the involvement of the Hedgehog and PI3K/Akt/mTOR pathways in leukemias, with a focus on their crosstalk and impact on cell death mechanisms.

## 2. The Signal Transduction Pathways

### 2.1. Hedgehog Signaling Pathway

The Hedgehog pathway (Hh) signaling cascade, a well-conserved pathway, is essential in both embryonic development and adult tissue homeostasis in species from insects to humans; it governs a range of cellular activities such as cell growth, development of distinct tissue types, regeneration of normal tissue, and regulation of both normal and cancerous stem cells. This pathway is characterized by mammalian ligands: Sonic Hedgehog (Shh), Indian Hedgehog (Ihh), and Desert Hedgehog (Dhh). Shh, the most expressed ligand, exerts important functions in different tissues, Ihh is involved mainly in skeletal development, while Dhh acts mainly in the development of the male gonads, the peripheral nerves, and the endothelium of large vessels [1,2]. The main events that occur in the signaling cascade are here examined. In the absence of one of the Hh ligands, the receptor Patched (Ptch) receptor, a 12-pass transmembrane protein localized in the primary cilium of the cell surface, inhibits signaling by suppressing the activation of smoothened (Smo), a seven-pass transmembrane protein belonging to the G protein-coupled receptor (GPCR) superfamily, which in turn exerts its effect on suppressor of fused (SUFU), a negative regulator of any Glioma-associated oncogene family (Gli) member. Gli interact with the N- and C- terminal domains of the SUFU, which are dragged in to enclose and contain it in a “sandwich-like” manner. Thus, a Gli member, stably bound and retained in the cytoplasm, could not translocate into the nucleus to act as a transcription factor [3]. The Gli transcription factor family includes three isoforms (Gli1, Gli2, Gli3) with highly conserved zinc finger DNA-binding domains, which represent the main effectors of this pathway. Gli proteins exist in various truncated or full-length forms [4]. The absence of Hh ligands induces proteolytic removal of the transcriptional activation domain in the C-terminus of Gli2/3, producing a shortened repressor form, Gli2/3R, which moves into the nucleus to inhibit Hh target genes, such as *Gli1*, *Ptch*, *Cyclin D1*, *Cyclin-dependent kinases (CDK)*, *c-Myc*, *Forkhead box-C2 (FOXC2)*, *B-cell leukemia/lymphoma protein (BCL-2)*, involved in DNA damage repair, invasion and migration, proliferation, cell cycle, apoptosis, and other cellular processes. The full-length Gli2/3 proteins, instead, are retained in the cytoplasm by the SUFU and then degraded. Gli2 and Gli3 could thus act as either activators or repressors (Gli2/3A or Gli2/3R), and, since Gli2R is rapidly degraded and significantly less stable than Gli3R, Gli2 acts mainly as a transcriptional activator, while Gli3 acts as a repressor [5]. In the presence of Hh ligands, Gli2/3 activity is directly regulated by Smo signaling, whereas Gli1 is regulated by SUFU only after Hh pathway activation to amplify the Hh signaling regulation of Hh target gene transcription [6]. When Hh ligands are bound to Ptch1, the receptor from the primary cilium is internalized and directed to the lysosome for degradation, eliminating its inhibitory effects on Smo. The specific mechanism by which Ptch1 catalytically inhibits Smo remains unclear, but some observations are known to date: (i) Ptch1 seems to have a prokaryotic resistancenodulation–division (RND) family transporter-like protein structure, and (ii) it does not interact directly with Smo. Moreover, Ptch1 contains a sterol-sensing domain (SSD) likely conferring the ability to bind sterols. Studies suggest that its endogenous cargo may be sterol molecules that are transported across the cell membrane to limit Smo access to these molecules, which are necessary for its activation. Ptch1 could therefore exert its inhibitory effect on Smo by decreasing its accessibility to cholesterol. Cholesterol seems to be an endogenous regulator since it binds to the cysteine-rich domain (CRD) on the Smo receptor, inducing a conformational switch to an open and active conformation [7,8,9,10]. This type of activation is also induced by phosphorylation of its intracellular C-terminal domain performed by multiple kinases, including protein kinase A (PKA), casein kinase 1 (CK1), CK2, GPRK2, and atypical protein kinase C (aPKC). The phosphorylation seems to be one of the most important post-translational modifications which characterizes Hh members [11]; cell ciliary localization of Smo is also important for its activation and is further regulated by SUMOylation. This type of modifications to Smo controls its trafficking to primary cilium, which is required for Hh signaling, and counteracts the ubiquitination process, which instead causes degradation via lysosome- and proteasome-mediated pathways [12]. The status of Smo activity influences Gli proteins and their fate. The post-translational modifications of Gli1/2/3 are the following: phosphorylation, ubiquitination, SUMOylation, acetylation, methylation, and O-GlcNAcylation. Direct phosphorylation by some protein kinases, such as PKA, PKC, mitogen-activated protein kinase 1 (MEKK1), glycogen synthase kinase 3 (GSK3), ribosomal S6 kinase beta-1 (S6K1), CK1, dual specificity tyrosine phosphorylation regulated kinase 1 (DIRK1), and Unc-51-like kinase 3 (ULK3), on Gli members positively regulates their activity, promoting their nuclear localization [13]. The signaling cascade here examined represents a canonical way of transmission of Hh signaling. In contrast, noncanonical activation of the Hh signaling pathway occurs when one of the components of the pathway is mutated or when not all typical components are involved. In the absence of the Hh, Ptch transmits signals from different pathways, such as PI3K/Akt/mTOR, Ras/Raf/MEK/ERK, Wnt/β-catenin, Notch, or Hippo. Noncanonical activation bypasses the Ptch–Smo complex, inducing the activation of Gli transcription factors downstream of Hh by other signaling pathways, such as PI3K/Akt/mTOR or other proteins, such as PKA, CK1, S6K1, ULK3, AMP-activated protein kinase (AMPK), GSK3β, or extracellular signal-regulated kinase (ERK2) [14]. Among these, one of the most discussed and examined is PI3K/Akt/mTOR, whose role in relation to Hedgehog signaling is explored in this review.

### 2.2. PI3K/Akt/mTOR Signaling Pathway

The PI3K/Akt/mTOR signaling pathway is highly conserved by evolution in all eukaryotic cells [15,16]. The main molecules involved in the signaling pathway are phosphatidylinositol 3-kinase (PI3K), protein kinase B (Akt), and mammalian target of rapamycin (mTOR). PI3K family proteins represent a large group of lipid kinases, grouped into three classes based on their structure, regulatory mechanism, and substrate specificities [17]. Each class is characterized by different isoforms of the catalytic and regulatory subunits. The class-I PI3Ks consist of a regulatory subunit p85, which binds to the receptors, and a catalytic p110 subunit, and this class is the most important class involved in cancer. The subunit p110 is encoded in four different isoforms, α, β, δ (subclass IA), and γ (subclass IB), and the first three are associated with p85, while p110γ is associated with other regulatory subunits, such as p101 and p84/p87 [18]. Class-II and class-III PI3Ks exert indirect effects in signaling regulation, since they have a role in membrane trafficking processes, maturation, autophagosome formation, autophagy flux, and cytokinesis. After a stimulus from the extracellular environment, receptor tyrosine kinases (RTKs), such as G protein-coupled receptor, platelet-derived growth factor receptor (PDGF-R), insulin-like growth factor-I receptor (IGF-IR), and Fms-related tyrosine kinase 3 (FLT3), recruit PI3K, which activate and catalyze the phosphorylation of the substrate phosphatidylinositol-4, 5-bisphosphate (PIP_2_) to product the second messenger phosphatidylinositol-3, 4, 5-trisphosphate (PIP_3_). This process causes the translocation of Akt from the cytoplasm to the membrane, promoting its activation by phosphorylation. The PI3K pathway could be negatively regulated by a lipid phosphatase and tensin homolog (PTEN), which dephosphorylates PIP_3_, preventing Akt activation. Akt is a serine/threonine kinase and in eukaryotes has three isoforms, Akt1, Akt2, and Akt3. Akt1 is found in all tissues, acts as a proto-oncogene, and is involved in various cellular processes, such as inhibition of apoptosis and induction of cell survival. Akt2 is mostly located in muscle tissue and fat cells, and it has a role in glucose homeostasis. Akt3 is usually expressed in the brain, in which its role and function have not been fully elucidated [19]. Full activation of Akt requires two phosphorylation in a row: the first one on T308 by PDK1 and the second one on S473 by mTOR. Akt exerts its antiapoptotic and pro-survival effects by the following functions: (i) phosphorylating and thus blocking pro-apoptotic BCL-2, Bcl-2 associated X protein (BAX), Bcl-2 associated agonist of cell death (BAD), and caspase 9 proteins; (ii) influencing some transcription factors, such as cyclic AMP-responsive element-binding protein (CREB), inhibitor of nuclear factor-κB (IκB) kinase (IKK) and a regulator of the survival factor, nuclear factor kappa-light-chain-enhancer of activated B cells (NFkB), (iii) increasing Mouse double minute 2 homolog (MDM2, negative regulator of p53) phosphorylation and consequently the inhibition of p53, (iv) fine tuning the function of Chk1, a cyclin-dependent kinase inhibitor (p21 and p27), modifying its phosphorylation state at the beginning of the cell cycle. Akt promotes cell proliferation, phosphorylating and inactivating the Forkhead box protein O1 (FOXO) transcription factor family and GSK3 protein [20]. Akt downstream phosphorylates and activates mTOR, a serine/threonine kinase protein included in two different protein complexes, mammalian target of rapamycin complex 1 (mTORC1) and mTORC2. Both protein complexes could be adjusted by nutrients such as lipid and cholesterol, mitochondrial functionality, and growth factors such as epidermal growth factor (EGF) and IGF [21]. mTORC1 (i) increases protein synthesis activating S6K1, (ii) regulates protein degradation inactivating eukaryotic translation initiation factor 4E-binding protein 1 (4E-BP1), a negative regulator of 5′cap-dependent mRNA translation, which dissociates from eIF4E, the eukaryotic translation initiation factor 4E, (iii) induces lipid biogenesis by activating sterol regulatory element-binding transcription factor 1 (SREBP1) and peroxisome proliferator-activated receptor γ (PPARγ) transcription factors, and iv) inhibits autophagy by blocking ULK1 [22]. Other targets of mTORC1 are signal transducer and activator of transcription 3 (STAT3), eukaryotic elongation factor 2 (eEF2), and retinoblastoma factor (Rb), important factors involved in cell growth, proliferation, metabolism, and migration. mTORC2 regulates actin cytoskeleton polarization via protein kinase C (PKC) α/β. Also mentioned among its substrates are IGF-IR and insulin receptor (Ins-R) [21,23].

The Hh and PI3K/Akt pathways are closely linked to proliferation and consequently to cell cycle regulation: Gli1 factor promotes the expression of genes involved in cell cycle progression while the PI3K/Akt signaling activates signals that promote cell survival, growth, and inhibition of apoptosis. Specifically, Gli proteins influence key processes such as progression in the cell cycle and cell division by promoting the expression of target genes, including cyclins (such as cyclin D and E) and cyclin-dependent kinases (CDKs), which regulate the G1/S transition. In addition, Hedgehog can inhibit the expression of cell cycle inhibitors, such as p21 and p27, promoting proliferation. This dysregulation is often observed in cancers, including leukemia, where overactivation of Hh contributes to the uncontrolled growth of malignant cells.

## 3. The Role of Hedgehog Signaling in the Cell Cycle

Different studies have shown that silencing Gli1 attenuates the Hh signaling pathway and is able to arrest the G2/M phase of cell cycle progression by downregulation of cyclin B and Rb phosphorylation, inducing apoptosis and autophagy [24]. Treatment with GANT61 in the T-ALL sensitive cell line showed that inhibition of Gli1 leads to cell cycle arrest. A previous study reported that Gli factors can upregulate FOXC1 expression, leading to the stabilization of Gli1/2 proteins through the suppression of their ubiquitination [25]. Other studies have also shown that GANT61, by blocking Gli functions, represses cell proliferation in several tumors, making it a good candidate for the treatment of hematological malignancies, including myelodysplastic syndromes and acute myeloid leukemia (AML), where treatment with GANT61 blocks the cell cycle in the G1/G0 phase in a dose- and time-dependent manner [26]. In HL-60 acute myeloid leukemia cell line, cyclopamine (Smo inhibitor) induced apoptotic cell death, cell cycle arrest in the G0/G1 phase, and monocytic differentiation (a strategy often used for the treatment of hematological malignancies), also causing downregulation of pAkt and ERK1/2, involved in anti-proliferative activity [27]. Several works have demonstrated the interaction between the two pathways, in which Gli1 accelerates mitosis by activating the PI3K/Akt/GSK3/CDK pathway. In this context, Gli1 overexpression increases the protein levels of GSK3α/β, cyclin D (cyclin D2 and D3), CDK4, and CDK6, inducing proliferation, drug sensitivity, and cell cycle progression. These data suggest that Gli1 is an upstream regulator of the Gli1-PI3K/Akt axis [25,26]. Synergism between Gli1 knock-down and Ara-c treatment leads to cell cycle arrest in the S phase, inhibits DNA polymerase, and directly regulates the CDK4 and cyclin D1 (CCND1) cell cycle genes, thus blocking the cell cycle in G1/S phase. Gli1 has been shown to regulate the phosphorylation of PI3K and Akt but not their expression level, while inhibitors of PI3K and Akt reduce the expression of Gli1 [28].

Aberrant signaling pathways drive enhanced proliferation of leukemic cells by disrupting cell cycle checkpoint control, leading to clonal expansion. Dysregulation of them can thus interfere by hindering DNA repair processes and promoting the accumulation of genetic abnormalities typical of leukemias, making both pathways key targets for targeted therapeutic strategies.

## 4. Hedgehog Pathway Alterations in Leukemic Malignancies

The first correlation between Hh signaling and hematopoiesis was reported two decades ago. Some relevant observations showed that Hh signaling may induce the expansion and increase of functional activity in hematopoietic stem cells (HSCs); both ligands Shh and Ihh, with other morphogenic signals, affect the development and maturation of T and B lymphocytes [28,29]. HSCs express Hh pathway genes, such as Ptch1, Smo, and Gli, ref. [30], while Hh ligands are expressed by stromal cells in the bone marrow and function as paracrine or autocrine survival signals. It is interesting to note that the mutations in different Hh pathway members are associated with the pathogenesis of hematologic malignancies such as chronic myeloid leukemia (CML), acute myeloid leukemia (AML), and acute lymphoblastic leukemia (ALL) [31,32]. Hh seems to stimulate tumor growth because its inhibition decreases it, and it is often associated with inactivating mutations of Ptch or activating mutations of Smo. Gli transcription factors, the main effectors of the pathway, could also be responsible for tumor expansion when mutated or altered in expression [29]. The main alterations of Hh signaling in leukemias are indicated in Table 1.

### 4.1. Hedgehog Pathway Alterations in Acute Lymphoblastic Leukemia (ALL)

ALL is caused by the neoplastic transformation of hematopoietic B cell or T cell progenitors and their spread into the bone marrow and bloodstream. ALL is the most common pediatric cancer, a heterogeneous disease, since B cell precursors may or may not be characterized by the presence of the Philadelphia chromosome [33]. It has been shown that upregulation of Hh signaling in T-ALL cells may depend on genetic aberrations in critical components of the pathway, autocrine signaling, or ligand-independent noncanonical mechanisms. Dagklis and coworkers conducted sequencing experiments in which multiple single-nucleotide variants (SNVs) were identified when Smo was truncated in the C terminus and point mutations of Gli1, Gli3, and Ptch2 [34]. In another study, the same laboratory observed ectopic expression of Shh and Ihh and upregulation of Gli1 in T-ALL patients and a decrease of T-ALL cell proliferation after inhibition of Smo and Gli1 [35]. Burns and colleagues. identified mutations in the gene pairs Ptch1/Gli2 or Gli1/SUFU, point mutations of Gli1, Gli2, Gli3, or SUFU, which seem to be associated with resistance to chemotherapy. Moreover, Ptch1 appeared to be the single most mutated gene within their patient cohort [36]. Taken together, these data indicate that the Hh pathway can be activated in different cases of T-ALL by mutations in critical proteins, representing an oncogenic pathway, and consequently a potential target for therapy in T-ALL.

### 4.2. Hedgehog Alterations in Acute Myeloid Leukemia (AML)

AML is a heterogeneous hematologic tumor that develops in stem cell precursors specifically belonging to the myeloid lineage [37]. The pathways involved, such as Hh signaling, are implicated in stem cell self-renewal and chemotherapy resistance, a characteristic feature of AML [38]. Studies have demonstrated the upregulation or altered expression of Hh components, such as Smo, Ptch1, and all three Gli transcription factors, in most AML samples. Chaudhry and coworkers focused on the role of Gli3R: since Gli3 was observed to be epigenetically silenced, the activation of the Hh pathway is caused by suppression of Gli3R rather than the regular interaction of Smo with an Hh ligand. This event could be the cause of resistance to SMO antagonists in most AMLs, and the restoration of Gli3R by hypomethylating agents may recover sensitivity to SMO inhibitors [39]. Wellbrock investigated the expression of Hh components and Gli1 inhibition by GANT61 or by gene silencing in AML cells both in vitro and in vivo. Gli1 inhibition has led to significant induction of apoptosis, reduced cell growth, and diminished colony formation in AML cells. Pathway activation appears to be triggered by the cellular microenvironment of AML blasts as high amounts of Hh ligands have been found in plasma, primary endothelial cells, osteoblasts, and bone marrow samples from AML patients compared with healthy individuals, supporting the conclusion that pathway activation could be caused by paracrine signaling from the microenvironment [40]. Different research groups have explored sensitivity to conventional chemotherapy such as anthracyclines (e.g., daunorubicin, idarubicin) and the nucleoside analogue cytarabine by the pharmacological and genetic inhibition of Hh, obtaining significant antileukemic effects against AML [31]. Another cause of resistance could consist in the association between increased levels of Gli1 and increased levels of UDP glucuronosyltransferase (UGT1A) enzymes, which inactivate drugs by glucuronidation (addition of glucuronic acid) in chemotherapy-resistant AML cells. Gli1 alone is sufficient to determine UGT1A-dependent cytarabine glucuronidation; thus, the inhibition of Gli1 by gene silencing or treatment with a Smo inhibitor (such as Vismodegib) sems to make AML cells mostly responsive to cytarabine [41,42].

### 4.3. Hedgehog Alterations in Chronic Myeloid Leukemia (CML)

CML is one of the most common leukemias in adults, though it is rare in children, and is characterized by the presence of the Philadelphia chromosome. Point mutations in the kinase domain of Bcr-Abl protein could lead to the resistance to tyrosine kinase inhibitor (TKI) therapy which characterizes this disease [43]. The Hh pathway represents one of the independent mechanisms required for leukemic stem cell (LSC) maintenance and a potential target for the treatment of CML, given that first- and second-generation TKIs (such as imatinib), targeting the Abl kinase ATP-binding domain, and third-generation drugs (such as ponatinib) targeting the specific T315I mutation of Bcr-Abl, are not always effective against CML [44]. Studies have demonstrated that cyclopamine (an inhibitor of Smo), and other Smo antagonists can limit LSC expansion and resistance to TKIs. Dierks and Zhao, in two different works, have demonstrated the importance of the role of Smo in CML in in vitro and in vivo models. Zhao showed that loss of Smo slowed the renewal of hematopoietic stem cells, reducing the transformation of CML cells [45], while Dierks showed that pharmacological inhibition of Smo in CML patients could help to reduce the spread of imatinib-resistant LSCs during treatment [46]. In addition to expression of Shh, Ptch1, and Gli1, BCL-2 levels are also upregulated in CML cell lines, and the inhibition of both Shh and BCL-2 could make leukemic cells more sensitive to imatinib [30].

### 4.4. Hedgehog Alterations in Chronic Lymphocytic Leukemia (CLL)

CLL is characterized by an overproduction of neoplastic B lymphocytes and their slow progressive spread in the bloodstream, bone marrow, and lymphoid tissues. It does not yet have a cure, in most cases. The key factor of this disease is represented by altered signaling via BCR, upregulation of anti-apoptotic factors, and communication between CLL cells and the microenvironment [47]. The role of the Hh pathway in CLL seems to be contradictory compared to other leukemias. Hegde et al. described the increased expression of Gli1, Gli2, SUFU, and BCL-2 in primary CLL cells acting as survival factors. Pharmacological inhibition of the Hh pathway suppressed pro-survival signals from the stromal microenvironment of CLL cells. Furthermore, selective downregulation of Gli1 decreased expression of BCL-2, suggesting that Gli1 regulates CLL cell survival through BCL-2, affecting the apoptotic process [48]. Decker and Ghia observed that in CLL samples, Hh signaling activation was associated with early disease progression and increased sensitivity to Gli1 inhibition, which seems to be more effective when compared to SMO inhibitors in overcoming stroma-mediated protective effects [49,50]. Indeed, Desch and colleagues demonstrated that Smo and Gli1 seem not to be differentially expressed in CLL cells when compared to normal B cells. They also observed that Smo inhibition alone did not influence CLL cell survival, while the Gli inhibitor GANT61 caused significant apoptosis in CLL cells but not in normal B cells, suggesting that expression of the transcription factor Gli1, a key element of the Hedgehog (Hh) pathway, is thus regulated by several pathways, such as the PI3K/Akt, RAS/RAF/MEK/ERK, Wnt/β-catenin, Notch or Hippo pathways, that act upstream through post-translational mechanisms, indirectly modulating the expression of genes critical for cell proliferation and survival [51]. Among the pathways whose action is most impactful on hematologic diseases, of greatest importance is PI3K/Akt, whose involvement in leukemic diseases has been extensively investigated. Here, we highlight key studies that support the role of the PI3K/Akt/mTOR pathway, one of the two pathways discussed in this review.

## 5. PI3K/Akt/mTOR Pathway Alterations in Leukemic Malignancies

The PI3K/Akt/mTOR pathway is one of the most altered pathways in cancer. Multiple genetic modifications in one of its key components could cause an uncontrolled hyperactivation, which leads to oncogenesis [52]. The main alterations of PI3K/Akt/mTOR in leukemias are collected in Table 2.

### 5.1. PI3K/Akt/mTOR Pathway Alterations in T-ALL

The most critical aspect of T-ALL is represented by constitutive activation of the PI3K/Akt pathway, caused, in most cases, by deletions or loss-of-function mutations in its negative regulator, PTEN, which cluster in exon 7 and cause protein truncation at the C terminus, with its consequent degradation. PTEN alterations seem to be more frequent than gain-of-function mutations in the regulatory (p85) and catalytic (p110) subunits of PI3K, PI3KCA (gene encoding for catalytic subunit p110α), or PI3KR1 (gene encoding for regulatory subunit p85α) and mutations in Akt1 [53]. Gutierrez et al. identified modifications in the PTEN, PI3K, and Akt genes. This laboratory identified the truncation of the C2 domain (membrane phospholipid-binding) clustering in exon 7 without altering the phosphatase containing the active site [54]. Two different post-translational mechanisms affecting the activity of PTEN, but not its structure, could be (i) overexpression of serine/threonine kinase CK2, which causes hyperphosphorylation of its C terminus, reduction of phosphatase activity, and stabilization of protein, which is therefore not degraded or (ii) high levels of reactive oxygen species (ROS), which are able to decrease PTEN activity. Both CK2 inhibitors and ROS scavengers could restore PTEN activity in T-ALL cells [55].

### 5.2. PI3K/Akt/mTOR Signaling Pathway Alterations in B-ALL

B-ALL is more common in children than in adults, but adults are at a higher risk of relapse because of a typical molecular factor, the Philadelphia chromosome (Ph). The subtype of B-ALL that expresses the fusion protein Bcr-Abl activates different signaling pathways, including PI3K/Akt/mTOR. Both in Ph+ B-ALL and Ph- B-ALL, PTEN is expressed, usually unmutated, unlike in T-ALL, but functionally inactivated because of the action of CK2. The upregulation of PI3K/Akt/mTOR in all cases of Ph+ B-ALL could also be dependent on STAT5 activation, which modulates the transcription of p85 and p110 subunits of PI3K and could act as a pro-survival factor [56]. In Ph- B-ALL, PI3K/Akt/mTOR regulation could be dependent on the constitutive activation of pre-B cell receptor (pre-BCR) or its non-expression or the presence of interleukin-7 receptor (IL-7R) mutations, as in T-ALL [57]. Holz et al., investigating the effects of combined BCL-2 and PI3K/Akt inhibition by perifosine and venetoclax, respectively, observed a synergistic action on different points of the apoptotic process and an antileukemic activity in B-ALL cell lines [58].

### 5.3. PI3K/Akt/mTOR Signaling Pathway Alterations in CLL

Some molecular modifications in CLL, such as somatic mutations in immunoglobulin (Ig)-heavy chain variable gene segments (IGHVs), are associated with the function of BCR [59]. BCR signaling is transmitted to multiple kinases, such as BTK and PI3K. Isoform PI3Kδ, the most critical in CLL, transmits pro-proliferative and pro-survival signals by Akt and mTOR and participates in other signaling derived from cell surface proteins including cluster-of-differentiation 40 (CD40), integrins, chemokine receptor type 4 (CXCR4), and other stromal factors. Inhibition of PI3Kδ by genetic or pharmacological inactivation results in reduced cell proliferation and migration [60]. A potent PI3Kδ inhibitor for CLL is idelalisib, which blocks the constitutive activation of the p110δ-dependent PI3K pathway, decreasing p-Akt, induces apoptosis by caspase activation, and inhibits stroma–CLL cell contacts, resulting in relevant anticancer activity [61]. Furthermore, it has been demonstrated that the inhibition of SH2-containing inositol5′-phosphatase (SHIP1), a protein with inhibitory action on PI3K signaling, leads to hyperactivation of Akt but also to elevated mitochondrial activity with subsequent oxidative stress due to ROS production that could cause lytic cell death [62].

### 5.4. PI3K/Akt/mTOR Signaling Pathway Alterations in AML

PI3K/Akt/mTOR signaling deregulation is present in AML. The main alterations of the pathway involve the overexpression of the p110δ subunit of PI3K, mTORC1 which is aberrantly activated, PTEN inactivation by CK2 and altered upstream factors, RTKs or GTPases [63]. Chen et al. have studied the correlation between FLT3 and PI3K signaling in FLT3-mutated AML cellular model (MV4–11 and MOLM13), in which PI3K signaling is hyperactivated as demonstrated when the pharmacological or gene silencing mTOR inhibition causes cell death. In FLT3-mutated AML cells, mTOR inhibition downregulates PI3K signaling, diminishing survival and showing its upstream influence in PI3K signaling [64]. FLT3 represents an important molecular target for the treatment of AML and FLT3 inhibitors have been developed and approved for clinical use [65]. Another factor that acts by PI3K signaling and may represent a new target in AML is programmed death ligand-1 (PD-L1), interacting with the PD-1. PD-L1 seems to be upregulated, and its knockdown caused inhibition of proliferation, increased apoptosis, and G2/M cell cycle arrest by downregulation of PI3K and p-Akt [66]. Since AML cells have increased mitochondrial mass and oxygen consumption, Hurrish et al. have observed that dual inhibitor of PI3K and histone deacetylase (HDAC), named CUDC-907, when used in combination with Venetoclax (BCL-2 inhibitor), suppressed oxidative phosphorylation and mitochondrial function, causing cell death in both cytarabine-sensitive and -resistant AML cells [67]. PI3K/Akt/mTOR in AML cells could also be activated by microenvironment-produced factors such as GPCR chemokine receptor type 4 (CXCR4), abundantly expressed on the leukemic cell surface, and its physiological ligand, produced by stromal cells, or β1 integrins on leukemic cells and stromal fibronectin, with upregulation of integrin-linked kinase-1 (ILK1), implicated in integrin-mediated signal transduction by Akt phosphorylation [68].

We explored the interaction between the Hh and PI3K/Akt pathways in leukemic diseases and showed that their alteration can significantly contribute to disease development. Hh appears in many cases to be activated in a noncanonical manner by some components of the PI3K/Akt pathway, showing the existence of interactions between elements of the two pathways and thus a crosstalk that is worth paying attention to in this context. Studies have shown that the PI3K/Akt pathway is able to enhance Gli1 activity through phosphorylation or stabilization mechanisms, often creating a reciprocal amplification loop, fueling not only the growth of cancer cells but also their ability to avoid therapies.

The combined inhibition of the two pathways parallels their pathological synergy and appears to be a promising approach for the treatment of leukemias, as has been observed in some of the studies discussed here.

## 6. Crosstalk Hedgehog Signaling and PI3K/Akt/mTOR Pathway

**Figure 1:**
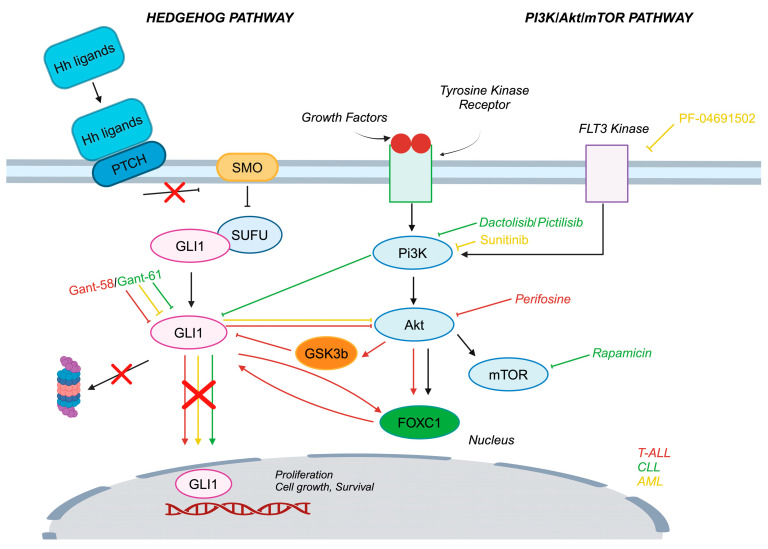
Crosstalk between the Hedgehog (Hh) and PI3K pathways in hematological malignancies. T-ALL: Red lines indicate a direct interaction between the two pathways. Inhibition of phosphorylated Akt downregulates Gli1 via GSK3ß, and dual inhibition of Akt and Gli1 synergistically induces cell death Additionally, FOXC1 and Gli1 form a positive feedback loop, where FOXC1 stabilizes Gli1 while Gli1 upregulates FOXC1 via Akt. CLL: green lines highlight a potential crosstalk between the two pathways. Dual inhibition of Gli1 and PI3K or mTOR leads to enhanced cell death compared to single-agent therapy, suggesting a cooperative effect. AML: yellow lines depict the interplay between the two pathways in AML. Triple inhibition of FLT3, PI3K, and GLI1 synergistically inhibits cell proliferation. Moreover, Gli1 inhibition downregulates Akt, sensitizing AML cells to targeted therapies. These findings suggest that targeting the crosstalk between the Hh and PI3K pathways may offer a promising therapeutic strategy for various hematological malignancies.

PI3K/Akt/mTOR interacts with the Hh pathway, inducing a non canonical activation. In detail, GSK-3β, CK1α and PKA phosphorylate Gli2/3 are proteolytically cleaved by β-transducin repeat-containing protein (β-Trcp) to produce the truncated repressor forms Gli2/3R. A possible mechanism for Gli1 is a likely phosphorylation by GSK-3β: hyperactivated Akt phosphorylates and inactivates GSK-3β, which does not induce proteasomal degradation of Gli1, stabilizing and activating it (thus promoting its transcriptional activity), even if a direct interaction between GSK-3β and Gli1 is not yet demonstrated [11,13,14,69]. What is remarkable is what has been observed in esophageal adenocarcinoma, in which S6K1 (downstream of mTOR)-phosphorylated Gli1 at S84 induces its translocation into the nucleus, according to a Smo-independent mechanism [70]. The crosstalk and the efficacy of the double inhibition of the Hh and PI3K/Akt pathways have however been examined in several types of cancer and diseases (endometrial hyperplasia, medulloblastoma, pancreatic cancer staminal cells, renal cell carcinoma, glioma, esophageal adenocarcinoma, colorectal, breast cancer and prostate cancer): it has been demonstrated that the synergistic effect of the inhibition of the two pathways reduces cell growth, survival, chemoresistance, and tumor progression [71,72,73,74,75,76,77,78,79]. In glioblastoma-initiating cells, for instance, a Smo inhibitor (NVP-LDE-225) and a PI3K/mTOR inhibitor (NVP-BEZ-235) exert a synergistic effect in the reduction of self-renewal capacity and progression of the cell cycle (suppression of cyclin D1). In addition, the treatment increases the expression of pro-apoptotic factors (cleaved caspase-3, cleaved PARP, and Bcl-2 interacting mediator of cell death (Bim)) and decreased anti-apoptotic markers (Bcl-2 and Bcl-xl) as well as the transcription factors involved in the mesenchymal–epithelial transition (EMT), such as Snail, Slug, and Zeb-1 [80]. The dual inhibition strategy of both pathways has also been studied also in leukemias. In T-ALL cells insensitive to Smo inhibition, downregulation of Gli1, enhanced by the inhibition of Akt phosphorylation, results in decreased cell proliferation and survival, further increased by pretreatment with an inhibitor of MEK/ERK signaling, demonstrating the importance of Hh interaction with other pathways [81]. Indeed, in T-ALL cells, a novel crosstalk between Gli proteins and FOXC1 has been identified: Gli factors could regulate the expression of FOXC1, which stabilizes them, weakening their ubiquitination. In T-ALL patients with central nervous system metastasis Gli1 deficiency has been observed: tumor spread was promoted by Hh activation, triggered via the Akt/FOCX1/Gli2 axis. Targeting the Akt/FOXC1 axis could decrease the activation of the oncogenic Hh pathway linked to Gli expression [25]. Gli also seems to be activated in a Smo-independent manner in CLL cells. Indeed, the involvement of the PI3K pathway and its interaction with Hh signaling promotes maintenance and survival. These findings once more confirm the importance of crosstalk and the double inhibition of Gli-Akt in hematological malignancies as a potential therapeutic strategy [82]. In AML cells with a mutated form of FLT3, a non canonical activation of Gli via FLT3 and PI3K has been found, since the inhibition of FLT3 and PI3K reduces Gli protein expression, whereas in AML cells of wildtype FLT3, Gli expression seems to be activated in a canonical manner [83]. Another in vitro study on AML cells showed that Gli inhibition caused a reduction of p-Akt, enhancing cell sensitivity to traditional chemotherapy and resulting in an antileukemic effect [84]. A schematic representation of the interactions between the two pathways described in leukemias is shown in Figure 1. The results from ex vivo studies and clinical trials involving different inhibitors of the Hedgehog and PI3K/Akt/mTOR pathways are shown in Table 3.

Gli proteins, central players in both canonical and non canonical Hh signaling, emerge as a crucial therapeutic target, as their high expression is associated with DNA repair, uncontrolled proliferation, and increased metastasis. Given the pivotal roles of these pathways, developing and testing dual inhibitors targeting both the Hh and PI3K/Akt/mTOR pathways offers a promising future strategy for overcoming therapy resistance and improving treatment outcomes in leukemia and other cancers.

As mentioned above, apoptosis is critical in preventing neoplastic transformation. The Hedgehog and PI3K/Akt pathways play key roles in regulating this process. In particular, the Hedgehog pathway modulates apoptosis through Gli-mediated transcriptional control of anti-apoptotic proteins such as Bcl-2. Meanwhile, the PI3K/Akt pathway modulates apoptosis via a cascade of phosphorylation that controls key pro-apoptotic proteins, including Bad and Caspase-9. Hh and PI3K/Akt are relevant targets for combination therapeutic strategies aimed at overcoming the resistance to apoptosis that often characterizes hematologic diseases, and their role is extensively investigated.

## 7. Involvement of Hh and PI3K/Akt/mTOR in Apoptosis in Leukemia Malignancies

Apoptosis or programmed cell death is a process that controls the development of the organism, tissue homeostasis, regulation of immune function, tumor suppression, and resistance to infection and leads to morphological changes that require activation of biochemical mechanisms [98]. Mitochondria play a key role in several cellular pathways, including apoptosis and induction of cell death. The intrinsic apoptosis pathway is governed by the BCL-2 family of proteins, which includes finely tuned pro-apoptotic and anti-apoptotic members. The pro-apoptotic effectors BAK and BAX are essential for initiating apoptosis, and their activation triggers pore formation in the mitochondrial outer membrane. This process results in the release of cytochrome c, a pro-apoptotic factor, from the mitochondrial intermembrane space into the cytosol, which promotes apoptosome assembly and caspase 9 activation, ultimately leading to cell death [99,100]. Modulation of mitochondrial activity represents a therapeutic strategy that blocks oxidative phosphorylation and releases pro-apoptotic proteins, such as cytochrome c, the BCL-2 family, BAK, and BAX, proteins responsible for regulating pro- and anti-apoptotic effects. The BAX/BCL-2 ratio is an important parameter in acute leukemia, and overexpression of the BCL-2 family of proteins is a mechanism of resistance to chemotherapy [25]. Leukemia cells, by mechanisms such as overexpression of antiapoptotic proteins (Bcl-2, Mcl-1, and Bcl-xL) or loss of expression of pro-apoptotic proteins (BAK/BAX), are able to evade the activation of apoptotic processes, favoring their survival during antileukemic therapies [100]. Evasion of the apoptotic process represents a mechanism of clinical resistance to therapies in leukemia [101]. Gli1, a downstream effector of Hedgehog signaling, has been implicated in the transcriptional regulation of Wnt5A and ROR1. Gli1 inhibitors, such as GANT61, and agents with anti-Gli1 activity, such as arsenic trioxide, have been shown to be effective in inducing the apoptosis of CLL cells. Some work has shown that arsenic trioxide can inhibit Gli1 transcription. By disrupting Gli1 regulation mediated by Wnt5A and ROR1, arsenic trioxide can sensitize CLL cells to apoptosis and overcome treatment resistance [102]. In a study by Neri et al., conducted on both B-pre-ALL cell lines and adult primary cells, RAD001 (everolimus), an mTORC1 inhibitor, used in combination with MK-2206 (an allosteric Akt inhibitor) or CCI-779 (another mTORC1 inhibitor) with GSK 690693 (an ATP-competitive Akt inhibitor), showed synergistic effects on the reduction of cell viability, cell cycle arrest in G0/G1, and induction of apoptosis and autophagy compared with the action of the individual drugs. These results demonstrate how the dual inhibition of Akt and mTOR could prove to be a promising therapeutic strategy, since hyperactivation of the PI3K/Akt/mTOR pathway represents a hallmark of ALL contributing to poor clinical outcomes [103]. The Sprouty (SPRY) family consists of four members, SPRY1, SPRY2, SPRY3, and SPRY4, which differ in tissue distribution, activity, and interaction partners. SPRY1 in particular is involved in the proliferation, differentiation, migration, and apoptosis of cells and is significantly upregulated in AML. The correlation between SPRY expression and the Hedgehog pathway in AML was demonstrated in a work by Guiyang Lv, in which the overexpression of SPRY1 significantly activated the Hedgehog pathway in AML cells. Indeed, silencing SPRY induced overexpression of Gli1 in HL-60 cells [104]. In vitro models of AML-FLT3-ITD have shown that three pivotal effectors of the Hh signaling pathway—Gli2, c-Myc, and p53—play key roles in disease pathogenesis. Studies on the underlying mechanisms have shown that triptonide induces cell cycle arrest (G0/G1 phase) and apoptosis in a time- and dose-dependent manner. Notably, a low-dose triptonide treatment also led to a significant increase in apoptosis in MOLM-13 cells on day 5, suggesting that prolonged exposure to triptonide may be necessary for its full apoptotic effect. Treatment with triptonide can activate p53, inhibiting cell division and survival in response to various stress signals. In addition, p53 plays a central role in the maintenance and function of hematopoietic stem cells, and its aberrations are known to influence the progression, biology, and therapeutic response of AML [105].

The Hh and PI3K/Akt pathways also significantly influence autophagy regulation. Hh, via Gli factors, promotes the expression of genes that modulate autophagy, and the precise relationship between Hh signaling and autophagy depends on the type of leukemia and the specific biological context. The PI3K/Akt pathway negatively regulates autophagy through the activation of mTOR, a key inhibitor of the autophagic process. For these reasons, they are often considered relevant targets for regulating autophagy in antileukemia therapies. However, in some conditions, autophagy does not represent a mechanism of cell death, but it could be activated to “safeguard” cancer cells from stress events, such as nutrient deficiency, hypoxia, or therapeutic treatments (e.g., chemotherapy or radiation therapy) as an adaptive response, thus promoting leukemia cell survival and chemoresistance. Because of its dual role, which is not always well defined in relation to tumor conditions, this process is often the subject of analysis, and we here investigated the correlation between Hedgehog, PI3K/Akt, and the autophagic process.

## 8. The Role of Hh and PI3K/Akt in Autophagy in Leukemia

**Figure 2:**
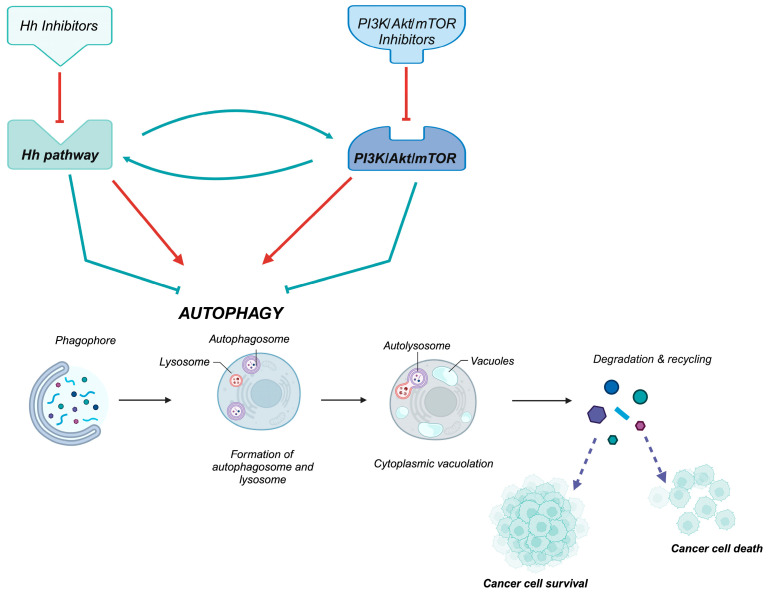
Role of Hedgehog (Hh) and PI3K/Akt/mTOR signaling in the regulation of the autophagic process in hematological diseases. Red lines indicate that inhibition of the Hedgehog or PI3K/AKT/mTOR pathways, via their respective inhibitors, activates the autophagic process. Green lines highlight the interaction between these pathways when active, demonstrating how they regulate each other to suppress autophagy. Autophagy plays a dual role in leukemic cells: it can enhance survival under stress, promoting chemoresistance through its cytoprotective effects, or drive cell death, contributing to tumor suppression.

Autophagy is a metabolic process of self-digestion, used by eukaryotic cells to recycle energy and eliminate misfolded proteins and damaged organelles; it is responsible for maintaining cellular homeostasis and enabling adaptation to change and stimuli arising from intracellular dynamics or the surrounding environment [24,106]. Induction of the autophagic process characterized by (1) the formation of autophagosomes, double-membrane vesicles which enclose proteins or organelles destined for degradation; (2) the fusion of autophagosomes with lysosomes; and (3) the degradation of the incorporated materials by lysosomal enzymes. LC3B (microtubule-associated protein 1A/1B-light chain 3 beta), is a marker of autophagy, which autophagosomes associate with and which sustains the post-translational modification that converts LC3I into LC3II, representing a hallmark of autophagy activation [107]. The involvement of the Hh pathway in the autophagic process can be defined as twofold: Hh signaling inhibits autophagy, and Hh signaling stimulates autophagy. The inhibition of autophagy by Hh signaling has been observed in both normal and cancerous cells, with regulation occurring through the Gli2-PERK-eIF2α pathway [108]. Inhibition of Hh signaling through overexpression of PTCH1 or PTCH2 induces an increase in the level of autophagic activity [106], while the Shh ligand has recently been proposed as a general positive metabolic regulator in cancer [109]: indeed, studies indicate that Shh-mediated activation of autophagy could influence cancer cell survival, thus becoming a new therapeutic target [110]. In cancer, the PI3K/Akt pathway plays a dual role in regulating autophagy, often promoting tumor progression or resistance to therapy. When activated, PI3K/Akt signaling stimulates a key autophagy suppressor, mTOR, which inhibits the initiation of autophagy by suppressing the ULK1 complex, which is involved in autophagosome formation, allowing tumor cells to grow and proliferate. Under stress conditions (nutrient deprivation, chemotherapy), inhibition of PI3K/Akt/mTOR can activate autophagy, promoting cancer cell survival by recycling intracellular components. This protective autophagy contributes to drug resistance in many cancers, and using the inhibition of PI3K/Akt to increase autophagic cell death is a promising therapeutic strategy in oncology [111]. The controversial role of autophagy in relation to the Hh pathway and PI3K/Akt signaling was examined in the Philadelphia-positive and imatinib (TKI inhibitor)-resistant CML cell lines Ar230 and K-562. Combined treatment with arsenic trioxide and interferon induced an antileukemic effect that was shown to be associated with increased autophagy due to significantly decreased expression of Gli1 and PTCH1 genes [112]. Chiarenza treated the CML cell lines K-562 and KU-812 with CUR61414 (Smo inhibitor), AT43 (Hh agonist), and MRTX, MRT94, 92, and 83 (Smo-specific inhibitory compounds derived from acylguanidine). They observed anti-proliferative activity with increased expression of BNIP3, an autophagy-related protein, and BCL-2 linked to the induction of apoptotic processes [113]. Zeng and coauthors [114] also showed that inhibition of Hh by Vismodegib (SMO inhibitor) in K562 and HL60 CML cells induced autophagy, visible in their increased autophagosome formation and autophagy flux. In this study, however, the inhibition of autophagy (by chloroquine or siRNA silencing of the autophagy-related ATG5 and ATG7 genes) significantly enhanced their cytotoxic effect on CML cells, indicating that autophagy acted as a survival mechanism. Combined inhibition of Hh signaling and autophagy thus led to a significant reduction in cell viability, proving to be an excellent therapeutic strategy. Investigating the underlying mechanism of action, Zeng et al. observed that combined inhibition (Hh and autophagy) in CML cells led to downregulation of the kinase activity of BCR-ABL oncoprotein and components of the PI3K/Akt/mTOR pathway, suggesting that Hh inhibition induced cytoprotective autophagy and downregulated the Pi3k/Akt pathway [114]. In AML cell lines, a synergistic effect on the proliferation reduction, cell cycle arrest, and activation of the autophagic process was observed. In MOLM-13 and CMK, the combination of PI3K and HDAC inhibitors induced an increase in LC3BII expression, corresponding to increased autophagy [115]. Cani and coworkers [116] examined the efficacy of the combination of three Akt inhibitors—perifosin, GSK690693 and MK-2206—in T-ALL. The findings showed that simultaneous triple inhibition of Akt exerted synergistic cytotoxic effects in Jurkat, Molt-4, CEM-S and CEM-R (T-ALL cell lines), inducing cell cycle arrest in the G0/G1 phase and apoptosis by caspase-3 activation and PARP fragmentation. Increased autophagy was also observed, suggesting a dual cytotoxic mechanism: blockade of autophagy by bafilomycin A1 increased the cytotoxicity of triple treatment, indicating the protective role of autophagy in T-ALL cells. Interestingly, the treatment also suppressed ERK pathway activity, highlighting the therapeutic potential of Akt targeting as a promising approach to counter drug resistance in T-ALL [116]. Luo’s study examined the antileukemic activity of AZD5363, an Akt inhibitor, in relation to the role of autophagy in T-ALL cells Jurkat, CCRF-CEM and PF382. AZD5363 not only induced apoptosis, arrested the cell cycle in G1 phase and inhibited cell viability, but also increased autophagosome formation, suggesting the activation of autophagy. Combined treatment of AZD5363 with hydroxychloroquine (HCQ), an autophagy inhibitor, significantly increased apoptosis and antileukemic effects by blocking autophagy, which was therefore shown to have a cytoprotective role [117]. Yuan et al. studied the role of overexpressed FAPP2 (phosphatidylinositol 4-phosphate adaptor protein 2) in T-ALL cells, and its knockout by shRNA induced autophagy. The mechanism involved the downregulation of key lipid signals (PI (4)P and PI (3,4,5) P3) and the inhibition of the PI3K/AKT/mTOR pathway. These outcomes suggest that FAPP2 might be a treatment target whose inhibition would offer enhancement of autophagy and suppression of leukemic cell proliferation [118]. In K-562 leukemic cells, at low concentrations, lycosin-I, a peptide derived from the venom of *Lycosa singorensis* (a spider species), was shown to induce apoptosis, ferroptosis through inhibition of PI3K/AKT/mTOR, and cell cycle arrest in the G1 phase. Increased LC3-II/LC3-I protein ratio and reduced p62 expression promoted augmented autophagy flux [119]. Also, in the NB4 cell line of acute promyelocytic leukemia (APL, a severe subtype of AML), increased expression of LC3B translated into increased autophagy induced by tanshinone IIa (TanIIa), a derivative of the *Salvia miltiorrhiza root*. This compound acted by suppressing the PI3K/Akt/mTOR axis and inducing the apoptotic process [120]. A study of thymosaponin A-III (TAIII, a saponin derived from *Anemarrhena asphodeloides*) has been shown to induce apoptosis and trigger autophagy by inhibition of PI3K/Akt/mTOR in T-ALL Jurkat cells. The induction of autophagy was demonstrated by the formation of autophagosomes and autophagic vacuoles observed by transmission electron microscopy and the expression of two autophagy-associated proteins, Beclin 1 and LC3-II. In addition, TAIII treatment increased BAX levels and reduced BCL-2 levels by suppressing the PI3K/Akt/mTOR signaling pathway, showing a new approach for T-ALL therapy. Figure 2 illustrates the role of the Hedgehog (Hh) pathway and PI3K/Akt/mTOR signaling in regulating autophagy in leukemias [121]. Since both PI3K/Akt and Hedgehog pathways are intrinsically linked to autophagy in different types of leukemia, a dual-inhibition strategy targeting both pathways could be a promising approach. An integrated approach, coupled with the precise modulation of autophagy, has the potential to markedly enhance the efficacy of antileukemia therapies, particularly if autophagy is meticulously regulated to preserve a fine balance. Such strategies merit further investigation [122].

## 9. Conclusions and Future Perspectives

Our contribution emphasizes the pivotal role of the complex crosstalk between the Hh signaling pathway and the PI3K/Akt/mTOR axis in the pathogenesis of leukemias and its involvement in cell death in these diseases. These two interconnected signaling networks govern cellular processes by fine-tuning the balance of growth versus the removal of unwanted cells. Dysregulation of these pathways contributes significantly to leukemic transformation and progression by promoting uncontrolled cell growth and evading apoptotic mechanisms. Moreover, aberrant activation of the Hh and PI3K/Akt/mTOR pathways can drive resistance to conventional therapies, with troublesome treatment outcomes. The consolidated knowledge that the two signaling pathways interplay suggests it would be worthwhile to develop innovative therapeutic approaches. Therapeutic strategies would indeed benefit from selective compounds capable of simultaneously targeting components of both pathways, hitting, from time to time, specific parts of it related to the pro-survival and/or pro-death processes. In addition, alterations, such as for example mutations, hyperactivation, and the lack of function of single enzymes of both pathways, may be identified as biomarkers predictive of the disease worsening, to better identify and stratify the patients who are the most likely to benefit from such targeted therapies.

## Figures and Tables

**Table 1:** Hedgehog pathway dysregulation in hematological malignancies. The Hedgehog (Hh) signaling pathway, a critical regulator of embryonic development and tissue homeostasis, has been implicated in the pathogenesis of various hematological malignancies. Aberrant activation of this pathway has been linked to the development and progression of acute lymphoblastic leukemia (ALL), acute myeloid leukemia (AML), chronic myeloid leukemia (CML), and chronic lymphocytic leukemia (CLL).

Hedgehog		Alteration
ALL	Mutations in critical protein	Gli 1/2/3 Ptch1/Gli2 SUFU Gli1/SUFU Smo truncated in C terminus Gli1/SUFU
AML	Chemotherapy resistance	Smo-Ptch1 Gli1/2/3 Gli1/UPD Glucoronosyltransferase
CML	Resistance tyrosine kinase inhibitors (TKIs)	Smo Shh upregulation Ptch1/gGli1 upregulation
CLL	Alteration of signaling and microenvironment	Gli1/2 SUFU

**Table 2:** Alterations in the PI3K/Akt/mTOR pathway in hematological malignancies. The PI3K/Akt/mTOR signaling pathway plays a crucial role in cell growth, proliferation, and survival. Aberrant activation of this pathway has been implicated in the pathogenesis of various hematological malignancies, including acute lymphoblastic leukemia (T-ALL and B-ALL), chronic myeloid leukemia (CML), and acute myeloid leukemia (AML). This table provides an overview of the alterations in key factors within this pathway that have been identified in these diseases.

PI3K/Akt/mTOR		Alterations
T-ALL	Deletion, mutations and activity	PTEN Akt1 P85/p110
B-ALL	Impaired function	PTEN P85/P110
CLL	Pro-survival signaling	Isoform PI3Kd
AML	Over-expression Aberrant activation Inactivation and mutations	P110d mTORC1 PTEN FLT3

**Table 3:** Ex-vivo studies and clinical trials in hematological malignancies. Ex vivo studies and clinical trials related to different leukemias are summarized in the table. The clinical trial column shows completed and ongoing trials in which inhibitors of the Hedgehog pathway or PI3K/Akt/mTOR have been or are being used.

Leukemias Findings Ex Vivo		Ref	Clinical Trials	Ref
T-ALL	Ectopic expression of Hh ligands and upregulation of Gli1	[35]	BEZ235 (dual inhibitor PI3K/mTOR) (ClinicalTrial.gov ID NCT01756118 completed) ONC201 (Akt/ERK inhibitor) (ClinicalTrial.gov ID NCT02392572 phase I)	[85]
	Mutations in Ptch1/Gli2 or Gli1/SUFU, point mutations in Gli1/2/3 or SUFU and Ptch1 associated with chemoresistance	[36]		
	Alterations of PTEN, PI3K, Akt: reduced therapeutic effects if phosphatase domain of PTEN is not mutated	[54]		
	Inactivated PTEN in primary cells: restore its activity	[55]		
	Gli proteins regulate expression of FOXC1 and in cases of central nervous system dissemination, Akt/FOXC1 triggers Hh signaling	[25]		
AML	Smo-independent activation of Hh signaling, Gli3 silenced, Gli3R inhibits AML growth through downregulation of Akt	[39]	Gilteritinib (FLT3 inhibitor) with venetoclax, decitabine, and cedazuridine (ClinicalTrial.gov ID NCT05010122 phase I/II)	[86,87,88,89,90,91]
	Hh activation due to paracrine signaling from microenvironment: high amounts of Hh ligands in plasma, primary endothelial cells, osteoblasts, and bone marrow samples	[40]	Gilteritinib with azacitidine (ClinicalTrial.gov ID NCT06022003 phase II)	
	Identification of FLT3 mutation in approximately 30% of patients and its association with poor prognosis	[65]	Gilteritinib with daunorubicin and cytarabine (ClinicalTrial.gov ID NCT05024552 phase I)	
	CUDC-907 (dual inhibitor of PI3K and HDAC) synergizes with venetoclax (BCL-2 inhibitor) to cause cell death	[67]	Gilteritinib with ivosidenib and enasidenib (ClinicalTrial.gov ID NCT05756777 phase I)	
	Overexpression of Gli1 increases Akt phosphorylation: Gli1 inhibition alone could improve drug sensitivity.	[85]	PF-04449913 (Smo inhibitor) with ARA-C, decitabine, daunorubicin, and cytarabine (ClinicalTrial.gov ID NCT01546038 completed)	
CML	Hh signaling activity supports neoplastic stem cells since constitutive active Smo increases stem cells number, promoting cancer progression	[45]	BEZ235 (dual inhibitor PI3K/mTOR) (ClinicalTrial.gov ID NCT01756118 completed)	[85]
	Upregulation of Smo induces Hh activation and chemoresistance in Leukemic Stem Cells (LSC)	[46]		
CLL	Gli1 regulates tumor progression by BCL-2	[48]	TGR-1202 (PI3K inhibitor) with ibrutinib (ClinicalTrial.gov ID NCT02268851 completed) Idelalisib (PI3K inhibitor) with rituximab (ClinicalTrial.gov ID NCT01539512 completed) Idelalisib with rituximab and bendamustine (ClinicalTrial.gov ID NCT01569295 completed)	[92,93,94,95,96,97]
	Activation of Hh pathway and overexpression of Gli1 factor associated with tumor progression	[49]		
	Constitutive Hh pathway activation due to high levels of Gli1 and Ptch1 transcripts and autocrine Dhh ligand secretion	[50]		
	CLL primary cells are sensitive to Gli inhibition but not to Smo antagonist: Gli activation independent of Smo	[51]		

## Data Availability

Not applicable.
